# The Medical Future

**Published:** 1920-12-04

**Authors:** 


### The Hospital

The Workers' Newspaper of Administrative Medicine and Institutional Life, Administration, National Insurance and Health.

No. 1800, Vol. LXIX. SATURDAY, DECEMBER 4, 1920. PRICE TWOPENCE

### THE MEDICAL FUTURE.

After the stormy criticisms and unbridled Press comments which have lately raged round the medical and institutional future in this country, it must come as something of a relief to the many earnest workers in a noble profession to hear some of the essential aspects of the case put forward in a dignified and reasoned manner at the recent meeting of the General Medical Council. It has appeared to us that quite unjustifiable popular confusion has been created between certain extensions of the existing Insurance Medical Service and between their immediate connection with the schemes outlined in the interim report of the Medical Consultative Council of the English Health Ministry, together with those coming from the corresponding body in Scotland. To state that these reports require the fullest consideration by the doctors and the public may now be a mere platitude, but, as Sir Donald Macalister in his Presidential address remarked, these proposals have for their object the better organisation of medical practice generally, the aim being to render more effective the manifold services which the profession of medicine in *all* its branches is capable of rendering to the nation. This statement, coming from so experienced a man of affairs, should tend to some considerable extent to bring back the focus of popular attention upon the real situation of the moment.

A scheme of health organisation, such as outlined in the Dawson Report, obviously cannot come into action without a wide revolution in medical practice. It implies both a change that begins at the very earliest days of a doctor's training, and a far better understanding on the part of the public of essentials in health and hygiene generally. Neither of these things can come immediately. To expect materialisation of so enormous a change in a few short months is indeed to dream of a medical Utopia, and if such an attitude were to lead in the near future to a general disappointment with the whole ideal of national health revision, the public would have themselves taken the first step towards placing insuperable difficulties in the way of the Ministry responsible.

Each question must be dealt with singly, carefully, and particularly from its simplest aspects upwards. It is therefore satisfactory that already, as we write, no little attention is being given to reconsideration of many sections of the now famous Miscellaneous Provisions Bill. Each health question thereby affected is to a greater or less extent in urgent need of review, and we trust that by the time these notes are published some degree of official agreement will have been reached, and without such drastic pruning of this measure as to negative even the first steps in the wholesale reforms it suggests.

But in popular discussion of these matters there is one factor not infrequently allowed to fall into the background. Ever to realise the ideal Medical Service we obviously need, generally speaking, a definitely modernised standard of professional training. This, again, cannot be achieved in less than a period of years. The consultant will still be needed, and will probably be of greater national worth as his experience is made available to numerically more people. But considerable modification is needed in the exact lines along which the great bulk of general practitioners are trained. That this fact, and many closely allied announcements of recent origin, are no new discoveries was also evidenced at the General Council meeting. Space forbids detailed reference to the recommendations and reports concerning the revised medical curriculum, but it is well to note that for many months past a large number of final examinations in medicine, surgery, and midwifery have been actually visited and inspected by the Council's representative. The same process has been applied to examinations in sanitary science for degrees and diplomas in public health. At the meeting itself Dr. John Yule Mackay, Chairman of the Council's Education Committee, introduced his report on professional education, in which a number of valuable opinions came to light, justifiably critical in parts, upon the existing examination system. The great danger appears to lie in the fact that to-day, in medicine, the exigencies of the examination threaten to dominate both teaching and study. This, however, is but one of many spheres to be dealt with, and the point is that the beginning is everywhere being made. On every hand is a vast amount of ground to be covered, and those men are needed who will not criticise only, but whole-heartedly help, without thought of self-interest, in a great national cause.

